# The Impact of Private Equity Hospital Acquisitions on Maternal Health for Medicaid Patients

**DOI:** 10.1111/1475-6773.70048

**Published:** 2025-10-04

**Authors:** Yang Amy Jiao

**Affiliations:** ^1^ Department of Health Management and Policy University School of Public Health Ann Arbor Michigan USA; ^2^ Bates White, LLC Washington DC USA

**Keywords:** labor and delivery, maternal health, Medicaid patients, private equity

## Abstract

**Objective:**

To examine the impact of private equity (PE) hospital acquisitions on maternal health for Medicaid patients.

**Study Setting and Design:**

This quasi‐experimental study focuses on 66 PE acquisitions of hospitals between 2014 and 2018, analyzing national Medicaid claims data from 2011 to 2020. Using a difference‐in‐differences (DiD) framework, the study compares labor and delivery (L&D) outcomes at PE‐acquired hospitals with matched control hospitals to evaluate the effects on patient volume, process of care, and quality outcomes for Medicaid patients.

**Data Sources and Analytic Sample:**

The analysis uses data from the Transformed Medicaid Statistical Information System (T‐MSIS) and Medicaid Analytic eXtract (MAX), including over 1 million L&D hospitalizations. The analytic sample comprises 66 PE hospitals and 290 matched control hospitals.

**Principal Findings:**

PE acquisition was associated with a significant 12% decrease in Medicaid L&D market share (*p* < 0.05). The reduction was more pronounced in states with larger Medicaid‐to‐commercial payment gaps (−15.8% vs. −7.2%). However, no significant changes were observed in low‐risk cesarean rates, number of procedures, length of stay, or severe maternal morbidity.

**Conclusions:**

PE acquisitions of hospitals are associated with reduced Medicaid market share, particularly in states with lower Medicaid reimbursement relative to commercial insurance. Policymakers should consider addressing these issues by adjusting Medicaid payment rates to support vulnerable populations in PE‐acquired hospitals.

**Summary**

- What is known on this topic
  ○ Private equity (PE) firms have significantly expanded their ownership stake in various healthcare sectors over the past two decades.
  ○ PE acquisitions of hospitals have been associated with changes in hospital behavior, including efforts to reduce costs and enhance operational efficiency.
  ○ Limited research has focused on the specific impact of PE acquisitions on the Medicaid population and maternal health outcomes.
- What this study adds
  ○ This study is the first to examine the effects of PE acquisitions on Medicaid‐covered labor and delivery hospitalizations, using Medicaid national claims data.
  ○ PE acquisitions were associated with a significant reduction in Medicaid labor and delivery hospitalizations, particularly in states with larger Medicaid‐to‐commercial payment gaps.
  ○ PE acquisitions were not associated with any changes in the process and quality of care for labor and delivery services for the Medicaid population.

## 1 Introduction

Private equity (PE) firms have significantly expanded their ownership stake in various healthcare sectors over the past two decades. From 2010 to 2020, PE firms invested $750 billion in the US healthcare industry [1], making it the second‐most popular target industry for PE after information technology (IT) [2]. The growing presence of PE firms in healthcare is evident across multiple provider types, including hospitals [3, 4, 5, 6, 7, 8, 9]. As of November 2023, at least 386 US hospitals are owned by PE firms, accounting for approximately 9% of all private hospitals and 30% of all for‐profit hospitals [10].

Prior studies have found evidence of consistently increased prices for privately insured patients, as well as increased utilization of care and higher volumes of encounters across multiple sectors [7, 8, 11, 12]. Studies of the impact on quality of care following PE investments in healthcare facilities have shown mixed results. Some studies have reported concerning findings associated with PE ownership, such as increased short‐term mortality [3], increased probability of having an ambulatory‐care‐sensitive (ACS) emergency department (ED) visit and ACS hospitalizations among Medicare patients in nursing homes [4], and increased hospital‐acquired adverse events among Medicare beneficiaries [13]. Conversely, other studies report moderate improvements in specific areas, such as reduced mortality among Medicare beneficiaries hospitalized with acute myocardial infarction, and increased clinic volume and In Vitro Fertilization (IVF) success rates in fertility clinics [14, 15]. La Forgia and Bodner attribute the positive effects to the fact that most patients are self‐pay, making the fertility clinic market a consumer‐centric healthcare setting with minimal market frictions and information asymmetries, thus incentivizing owners to invest in quality. The mixed results indicate that the effect of PE acquisition depends on the healthcare market characteristics and the patient population.

Medicaid enrollees, who are reimbursed at lower rates than Medicare or commercially insured patients, may be viewed as a less financially desirable patient population from the perspective of PE firms. In addition, Medicaid patients often face greater clinical and social complexity, making them more vulnerable to changes in healthcare management and patient selection practices. In 2021, Medicaid paid for approximately 41% of all births in the United States [16]. The significance of maternal health for the Medicaid population underscores the critical need to understand the effect of PE hospital acquisition on this demographic.

In this study, we estimated the impact of PE ownership of hospitals on maternal health outcomes of Medicaid enrollees using national claims data from the CMS Medicaid Analytic eXtract (MAX) and the Transformed Medicaid Statistical Information System (T‐MSIS) Analytic Files (TAF) from 2011 to 2020. Employing a generalized difference‐in‐differences framework, we compared two sets of outcomes in PE‐acquired hospitals and matched control hospitals: (1) patient volume and patient selection, and (2) process and quality of care.

## 2 Materials and Methods

### 2.1 Private Equity Acquisitions

Hospitals that were acquired by PE for the first time between 2014 and 2018 were included as treated hospitals. Hospitals acquired via primary or add‐on leveraged buyout between 2003 and 2018 are identified following the methods in previous studies on PE hospital acquisition [14, 17]. Hospitals that were acquired by PE before 2014 were excluded from the control hospitals, so the control hospitals were all never treated hospitals. The PE acquisition of Hospital Corporation of America (HCA) is not included in the analysis as it happened in 2006, and Medicaid claims data are only available starting in 2011. Following a previous study [14], critical access hospitals were excluded due to their smaller inpatient bed count, cost‐based payment structure, and fundamental differences in the local market. Because PE firms are likely to exit their investment between 3 and 7 years post acquisition [18], we excluded observations for acquired hospitals that were more than 4 years after acquisition. The final sample consisted of 66 PE hospitals and 290 matched control hospitals with 1,071,343 L&D inpatient hospitalizations.

### 2.2 Medicaid Claim Data

To link PE acquisitions to Medicaid claims, we constructed an annual crosswalk dataset linking National Provider Identifier (NPI) to the Center for Medicare & Medicaid Services (CMS) Certification Number (CCN), using Medicare inpatient claims from 2011 to 2020.

Inpatient claims from MAX from January 1, 2011, to December 31, 2015, and TAF from January 1, 2014, to December 31, 2020, were matched to PE acquisition data using NPIs. CMS required all states to transfer their data submission from MAX to T‐MSIS in 2014 and 2015. During this period, some hospitals suffered from reporting issues such as higher‐than‐normal missing diagnosis codes, procedure codes, and loss of provider information. PE‐acquired hospitals were excluded from the analysis if they had reporting issues in the acquisition year and the year prior (the definition of reporting issue and further exclusion criteria applied are included in eMethod 1). Hospital‐years with reporting issues in 2014 or 2015 were excluded from the study. Additionally, if, according to DQ (Data Quality) Atlas, one of the following areas, total Medicaid and CHIP Enrollment, claims volume—IP, and billing provider NPI–IP, was considered unusable or of high concern, records from that state‐year were excluded from the analysis.

### 2.3 Provider Data and American Community Survey Data

Facility names and Medicare provider numbers were used to link hospitals in the American Hospital Association (AHA) Annual Survey Database to obtain hospital‐level characteristics. Facility‐level characteristics include bed size, estimated full‐time personnel count, teaching status, sole community provider status, Medicare inpatient day share, Medicaid inpatient day share, core‐based statistical area designation, ownership status (for‐profit, non‐profit, or government‐run), and zip code. Hospital zip codes were used to link county‐level information from American Community Survey (ACS) data, such as median household income, percentage of population below the poverty line, and percentage of population with any Medicaid/means‐tested public health insurance coverage.

### 2.4 Definition of Outcomes

We identified over 16 million L&D hospitalizations using the delivery code in MAX or a combination of diagnosis codes, procedure codes, and DRG groups in TAF [19, 20, 21]. eTables S1 and S2 contain codes for L&D claim identification. We chose not to follow the technical specifications released by CMS because their primary purpose is to identify pregnant and postpartum beneficiaries, and postpartum and prenatal diagnosis and procedure codes are included [22, 23]. For the purpose of this study, only inpatient claims that involve L&D should be included.

The annual number of Medicaid‐covered L&D hospitalizations from 2011 to 2020 was benchmarked to the number of Medicaid‐covered deliveries in hospital settings from 2016 to 2020, with a matching rate ranging from 87.7 to 94.5 [24].

To measure the impact on patient volumes at PE hospitals, we constructed the outcome as the hospital's share of Medicaid L&D hospitalizations in a hospital referral region (HRR) for each hospital‐year. HRRs are defined using Dartmouth Atlas boundaries and remain constant over time [25]. The denominator includes all labor and delivery hospitalizations in the HRR in that year without any exclusion. Hospitals entering or exiting the market adjust the denominator accordingly. This measure is calculated by dividing the number of Medicaid L&D hospitalizations for each hospital‐year by the sum of all Medicaid L&D hospitalizations within that HRR for that year. This HRR share represents the proportion of Medicaid L&D hospitalizations associated with each hospital relative to other hospitals within an HRR market. As PE firms cannot impact the total number of Medicaid L&D hospitalizations in an HRR, any change in the share after PE acquisitions could indicate changes in the distribution of Medicaid intake within an HRR. Hospitals located in HRRs that had only one hospital at any point are excluded from the market share analyses. Hospitals with zero Medicaid L&D hospitalizations in a year have the HRR share equal to zero and are included in the analyses.

The effect of PE on patient volumes can depend on the relative prices between commercial patients and Medicaid patients, as it measures how much Medicaid pays compared to commercial insurers in each state. Relative price is defined as the ratio of state‐level weighted average Medicaid payment to the state‐level weighted average commercial payment using data from the Health Care Cost Institute (HCCI) [26]. Low relative prices suggest higher payment gaps and higher opportunity costs of having a Medicaid L&D hospitalization under capacity constraints. To examine whether the effect of PE varies depending on relative prices, we conducted subgroup analyses to examine the heterogeneity in the effect of PE acquisition on Medicaid patient volumes separately for hospitals in states with high relative Medicaid‐to‐commercial prices (top tercile) and those in states with low relative prices (bottom tercile).

For process and quality of care, we examined the probability of having a low‐risk cesarean birth, the number of procedures during the hospitalization, length of stay, and severe maternal morbidity (SMM) at the hospitalization level. Hospitalizations included in the numerators of the market share outcome were included in the analytic sample. Codes to identify low‐risk pregnancy, cesarean birth, and SMM are included in eTables S1, S3, and S4. The number of procedures and the length of stay were right winsorized at the 99th percentile (values above this point were set to the 99th percentile) to reduce the effect of extreme outliers.

### 2.5 Additional Exclusion Criteria

Hospitals without established maternity care units during pre‐trend periods (2011–2013) were excluded, with details about the exclusion criteria provided in eMethod S1. However, their numbers of hospitalizations were included in the denominator when calculating the share of Medicaid L&D hospitalizations in an HRR. For hospitalization level analysis, we further excluded enrollees older than 64 or who were dual eligible during the month of hospitalization. Less than 1% of admissions were excluded. eFigure S1 shows the flow diagram for inclusion and exclusion.

### 2.6 Statistical Models

We employed the difference‐in‐differences framework to compare outcomes in hospitals acquired by PE with those not acquired by PE before and after acquisitions. The non‐PE‐owned hospitals serve as counterfactual outcomes to represent what would have occurred to PE‐acquired hospitals in the absence of acquisitions. To address potential selection issues regarding which hospitals were acquired by PE, we used a matching method where each PE hospital was matched to up to 5 non‐PE hospitals without replacement, following prior PE studies [8, 13, 14, 17]. The matching algorithm required exact matches on binary variables (urban vs. rural, ownership status, and teaching status) and within 0.5 standard deviations for continuous covariates, including bed counts, personnel counts, county median household income, and county percentage of population below the poverty line. Those variables are selected as they might impact PE acquisition.

We adopted the doubly robust staggered difference‐in‐difference analysis developed by Callaway and Sant'Anna [27], which accounts for treatment heterogeneity by group (based on the timing of PE acquisition) and treatment time (time period relative to acquisition time). For sensitivity analyses, we conducted two‐way fixed‐effects (TWFE) models with standard errors clustered at the hospital level (with details in eMethod S2). Outcomes are assessed from 3 years before and up to 4 years after acquisition.

In the admission level analyses for quality and procedure of care, we further controlled for enrollees' age, race, and ethnicity (White, Black, Asian, Hispanic, and other/unknown), and whether the delivery involved a stillbirth in both Callaway and Sant'Anna models and TWFE models.

## 3 Results

The study sample consisted of 290 never‐PE‐acquired hospitals with 904,292 L&D hospitalizations and 66 hospitals that were acquired by PE firms with 173,788 L&D hospitalizations. Table 1 reports summary statistics in 2013 for PE‐acquired hospitals, matched controls, and all control hospitals, representing 1 year of the multi‐year panel. Most PE‐acquired hospitals in our sample were small‐sized (71.2%), for‐profit (68.2%), and located in metropolitan areas (54.6%). Additionally, 23.5% were teaching hospitals, and 21.2% were sole‐community‐provider designation by CMS. Examining L&D hospitalizations in 2013, the average age was 25.9 years in PE‐acquired and matched control hospitals. PE‐acquired hospitals had fewer Black mothers (14.6% vs. 20.2%) and Hispanic mothers (22.6% vs. 28.6%) and a lower C‐section rate (27.7% vs. 29.2%) at baseline. Among Medicaid‐covered admissions, PE‐acquired hospitals had a higher percentage (50.2% vs. 44.1%) of claims paid by FFS Medicaid.

**TABLE 1:** Baseline characteristics of hospitals and L&D hospitalizations in 2013.

	PE hospitals	Matched controls	All controls
*Panel A: Hospital level characteristics*			
No. of hospitals	66	290	2505
Ownership status, no. (%)			
For‐profit	45 (68.2)	188 (64.8)	258 (10.3)
Non‐profit	17 (25.8)	82 (28.3)	1766 (70.5)
Government‐run	4 (6.06)	20 (6.9)	481 (19.2)
Teaching hospital, no. (%)	16 (23.5)	76 (22.8)	919 (37.0)
Sole community provider, no. (%)	14 (21.2)	57 (19.6)	353 (14.1)
Hospital beds, mean (SD)	160.36 (107.2)	155.71 (103.0)	201.08 (169.7)
Hospital size, no. (%)			
< 100 beds	47 (71.2)	214 (73.8)	1585 (63.3)
100–199 beds	14 (21.2)	58 (20.0)	494 (19.7)
200–349 beds	4 (6.1)	17 (5.9)	236 (9.4)
≥ 350 beds	1 (1.5)	1 (0.3)	190 (7.6)
Employee FTE, mean (SD)	725.7 (585.3)	763.9 (611.6)	1344.5 (1707.0)
Medicare inpatient day share, % (SD)	42.1 (9.9)	39.0 (12.7)	39.5 (13.0)
Medicaid inpatient day share, % (SD)	13.0 (9.1)	11.4 (7.7)	12.7 (9.5)
CBSA designation, no. (%)			
Rural	2 (3.0)	9 (3.1)	309 (12.3)
Metropolitan	36 (54.6)	186 (64.1)	1655 (66.1)
Micropolitan	28 (42.4)	61 (21.0)	541 (21.6)
Median household income, mean (SD)	47569.3 (12859.7)	49944.3 (12679.6)	51486.8 (13194.6)
% of population with an income to poverty ratio under 1.00, mean (SD)	16.8 (5.3)	16.4 (5.3)	16.0 (5.6)
% of population with any Medicaid insurance coverage, mean (SD)	15.1 (4.7)	14.7 (4.6)	14.9 (5.5)
*Panel B: Hospitalization level characteristics*			
No. of L&D hospitalizations	24,094	111,557	1,005,038
Age, mean (SD)	25.9 (5.7)	25.9 (5.7)	26.6 (5.8)
Race and ethnicity, no. (%)			
Asian and Native American	1691 (7.0)	3581 (3.2)	57,657 (5.7)
Black	3526 (14.6)	22,563 (20.2)	216,034 (21.5)
Hispanic	5443 (22.6)	31,933 (28.6)	276,096 (27.5)
White	12,083 (50.2)	48,314 (43.3)	398,159 (39.6)
Other or unknown	1348 (5.6)	5166 (4.6)	57,092 (5.7)
C‐section, no. (%)	48,091 (27.7)	264,593 (29.3)	2,444,952 (27.7)
Claim paid by FFS Medicaid, no. (%)	12,103 (50.2)	49,159 (44.1)	493,396 (49.1)

*Note:* One PE hospital acquired in 2018 used AHA data from 2015, as 2013 data is not available.

Abbreviations: CBSA, core based statistical areas; C‐section, Cesarean section; FFS, fee for service; FTE, full‐time equivalent; L&D, labor and delivery; PE, private equity; SD, standard deviation.

### 3.1 PE Acquisitions' Effects on Patient Volumes and Patient Selection

Table 2 presents the aggregated treatment effects from the Callaway and Santa Anna estimation. Overall, we observed a 1.3 percentage point decrease in the share of Medicaid L&D hospitalizations, which accounts for 12% of the baseline average for PE hospitals. The right side of Table 2 shows the subgroup analysis by state‐level relative price. The overall relative price was 0.42 (row 5), indicating that, on average, Medicaid paid 42% of what commercial insurers paid for L&D services. In states with high relative prices, the average relative price was 0.56, compared to 0.30 in states with low relative prices, reflecting a substantial difference between the two groups. Notably, the magnitude of decrease was more pronounced in hospitals from low relative price states, with a reduction of 1.9 percentage points compared to 0.8 percentage points in hospitals from high relative price states.

**TABLE 2:** Changes in access for Medicaid enrollees associated with PE acquisition.

	Overall	High vs. low relative price	
		High	Low
PE acquisition effect	−0.013* [−0.023, −0.002]	−0.008 [−0.018, 0.003]	−0.019 [−0.039, 0.001]
Pre‐acquisition market share, mean (SD)	0.11 (0.14)	0.11 (0.11)	0.12 (0.17)
Relative price, mean (SD)	0.42 (0.14)	0.56 (0.13)	0.30 (0.05)
N	2808	476	1258

*Note:* The relative price is defined as the state‐level Medicaid‐to‐commercial price ratio. Hospitals in states with high relative prices (top tercile) and hospitals in states with low relative prices (bottom tercile) are included in the subgroup analyses. Coefficients are estimated using the Callaway & Sant'Anna method described in the Method. Standard errors are clustered at the hospital level. Brackets contain 95% confidence intervals. The average of pre‐acquisition market share for PE hospitals is included to aid in the interpretation of the magnitude.

Abbreviation: SD, standard deviation.

* Indicates *p* < 0.05.

**FIGURE 1:**
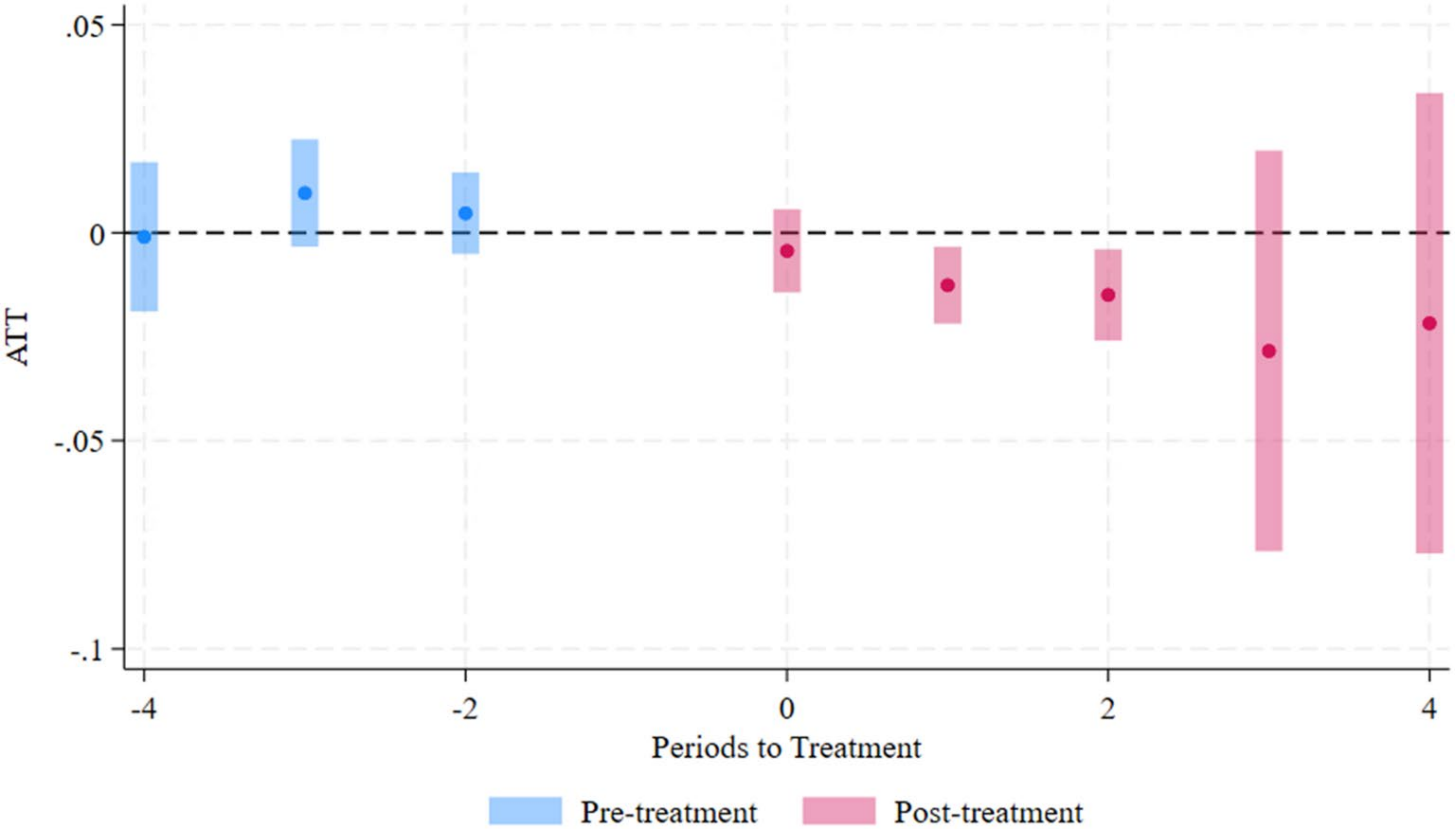
Longitudinal effects on share of Medicaid L&D hospitalizations in HRRs. *Note:* Pre‐treatment periods are shown in blue, and post‐treatment periods are shown in pink. The plot presents the average treatment effect on the treated (ATT) for each period relative to the year prior to the acquisition event. Dots represent point estimates, and the bars indicate 95% confidence intervals. Confidence intervals were computed using robust standard errors clustered at the hospital level. The widening of confidence intervals in later periods is due to the smaller sample size, as half of the PE acquisitions in the sample occurred in 2018.

Figure 1 displays the longitudinal coefficient plots for the share of Medicaid L&D hospitalizations. The conditional parallel trends are held in the pre‐treatment periods. The increased size of the confidence interval in post‐treatment periods three and four can be attributed to the decrease in sample size, as half of the PE acquisitions in the sample occurred in 2018, leaving only 2 years of follow‐up periods.

### 3.2 Subgroup Analysis by Ownership and Hospital Size

We observed a 1.1 percentage point decrease in the share of Medicaid L&D hospitalizations among small hospitals and a 1.7 percentage point decrease among large hospitals, with the decrease being roughly proportional to their respective PE baseline averages (eTable S5). Additionally, non‐profit hospitals had a larger HRR share at baseline (0.14 vs. 0.11). However, post‐acquisition non‐profit hospitals experienced a 15% decrease, compared to a 6% decrease among for‐profit hospitals.

### 3.3 Patient Selection

We further examined whether there was any patient selection by age and racial group post‐acquisition. It also sheds light on whether the decreased market share disproportionately impacted certain types of patients. Understanding patient selection behavior further helps in accurately assessing the impacts on process and quality of care. PE‐owned hospitals did not show any significant changes in patient race and age distribution post‐acquisition (eTable S6).

### 3.4 PE Acquisitions' Effects on Process and Quality of Care Outcomes

**FIGURE 2:**
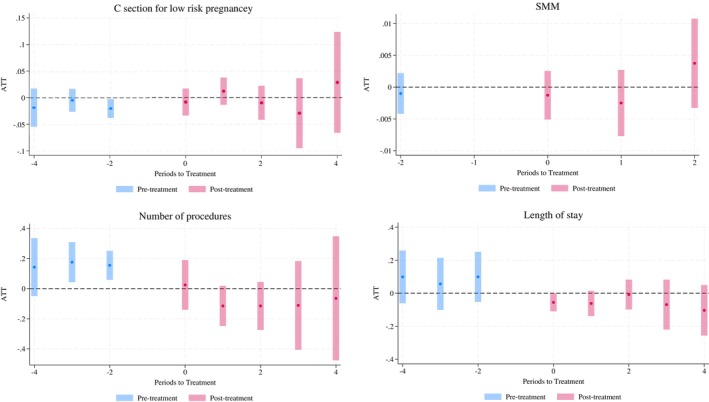
Longitudinal effects of PE acquisition on process and quality of care. *Note:* Pre‐treatment periods are shown in blue, and post‐treatment periods are shown in pink. These plots present the average treatment effect on the treated (ATT) for each period relative to the year prior to the acquisition event. Dots represent point estimates, and the bars indicate 95% confidence intervals. Confidence intervals were computed using robust standard errors clustered at the hospital level. SMM analysis only includes hospitals that were acquired in 2018, using data from 2016 to 2020.

Table 3 shows the results for process and quality of care. There was no statistically significant impact of PE acquisition on low‐risk cesarean birth, SMM, the number of procedures, or length of stay. Additionally, the magnitudes of the aggregated effects were not substantial. The effects of low‐risk C‐section and length of stay were precisely estimated with tight confidence intervals. For instance, the length of stay result can rule out any decrease greater than 0.13 per hospitalization, which is a 6% decrease. Figure 2 illustrates the longitudinal coefficients plot for each outcome. We conducted subgroup analyses based on maternal age (greater than 35 is considered advanced age) and race (white vs. non‐white) to see if any subgroups were affected differentially. Similar results were observed for low‐risk cesarean birth, the number of procedures, length of stay, and SMM per 1000 across both age and racial groups (eTable S7).

**TABLE 3:** Changes in process and quality measures associated with PE acquisition.

	Low risk C‐section	SMM per 1000^a^	No. procedures	Length of stay
PE acquisition effect	−0.002* [−0.037, 0.033]	0.020 [−4.754, 4.713]	−0.072 [−0.223, 0.079]	−0.057 [−0.13, 0.02]
Pre‐acquisition outcome, mean (SD)	0.26 (0.45)	1.42 (40.21)	2.99 (1.18)	2.33 (1.02)
*N*	1,035,295	426,912	1,106,414	1,106,414

Abbreviations: C‐section, Cesarean section; SD, standard deviation; SMM, severe maternal morbidity.

* Indicates *p* < 0.05.

^a^ Due to differences in data structure between MAX and TAF inpatient claims, it's important to note that MAX claims only contain 10 diagnosis codes (some states in earlier years only have 2), whereas TAF claims could include up to 13. As a result, the SMM rate is not directly comparable between earlier years in MAX and later years in TAF. Therefore, the SMM analysis only includes hospitals that were acquired in 2018 using data from 2016 to 2020. Coefficients are estimated using the Callaway & Sant'Anna method described in the Method. Standard errors are clustered at the hospital level. Brackets contain 95% confidence intervals. The average outcomes during the baseline period for PE hospitals are included to aid in the interpretation of the magnitude of the effects.

Sensitivity analysis results are included in eTables S8–S10. TWFE sensitivity analysis results are included in eTable S8. Findings were consistent when the outcome was the absolute number of Medicaid L&D hospitalizations rather than the HRR share (eTable S9). We used logit models for low‐risk cesarean birth, SMM, and patient selection models and found similar null results (eTable S10).

## 4 Discussion

Using a matched difference‐in‐differences approach, we found a 12% decrease in market share of Medicaid L&D services post PE acquisitions, which aligns with findings from Richard and Whaley's investigation, where they also report a 13% decrease in pregnancy‐related hospitalizations for the HCA PE acquisitions with no evidence of patient selection, changes in treatment intensity, or unfavorable selection of Medicaid enrollees [28]. Obstetrics and delivery services are among the leading money‐losing services offered by hospitals, which may contribute to this trend, as PE‐owned hospitals may face pressure from investors to avoid low‐profit services [29]. Since we only have access to Medicaid claims, we cannot confirm whether the decrease was specifically targeted at Medicaid enrollees or if PE hospitals strategically shifted away from obstetric care for all patients.

The subgroup analyses based on relative pricing show that in states where Medicaid paid less compared to the commercially insured, the decrease in market share is more pronounced. This suggests some evidence that Medicaid enrollees might be impacted disproportionately, particularly in states with lower Medicaid‐to‐commercial relative prices. Some Medicaid enrollees might need to deliver their babies at a less preferred hospital. The welfare implication is unclear as it depends on the reduced proximity to PE hospitals and the quality of care they receive at the alternative hospitals. Since most PE‐acquired hospitals are not located in rural areas, the decreased welfare due to reduced proximity to hospitals is likely to be less severe compared to cases where rural hospitals close their maternity units.

Both large and small hospitals show proportional decreases relative to their baseline HRR shares. Non‐profit hospitals had a higher HRR share at baseline, which is consistent with other studies showing non‐profit hospitals generally take more Medicaid patients [30, 31]. Most non‐profit PE hospitals in the analytical sample were converted to for‐profit hospitals by the end of the study period. The larger percentage decrease among non‐profit hospitals indicates an additional PE ownership effect beyond the shift to for‐profit status.

Despite previous studies finding a decrease in high‐cost patients in PE nursing homes and a shift in patient mix toward younger patients and fewer dually eligible beneficiaries in PE hospitals, we did not find any patient selection based on age and race [3, 13].

We found no significant effects of PE ownership on the low‐risk C‐section rate, despite prior studies indicating that financial and administrative incentives are associated with high rates of cesarean deliveries [32, 33, 34, 35, 36]. There are two possible explanations for these null results. First, higher payment for cesarean delivery does not always translate to higher profitability. Although both commercial payers and Medicaid reimburse about 50% more for cesarean deliveries compared to vaginal deliveries [26, 37], and there are administrative incentives such as greater ease in scheduling and better management of staff time and resources [38], cesarean deliveries are also costlier [39]. The profitability of cesarean deliveries for Medicaid enrollees varies at the hospital level due to different rates from states and specific hospital‐level costs. Moreover, several states offer payment incentives to lower the cesarean rate for Medicaid patients [40], further complicating the profitability of cesarean deliveries and making it challenging for researchers to measure.

Second, PE firms' acquisition of hospitals may not have a direct influence on physicians' day‐to‐day clinical practice. Physicians might not be directly employed by the hospital and instead be affiliated with practice groups that are not owned by PE. Prior PE literature with negative impacts on patients often attributes the results to cost‐cutting measures implemented by PE firms to maximize profitability [3, 13]. PE firms might lack the expertise needed to understand the clinical intricacies required for developing specialized strategies for each service. Even if they do, maternity units, sometimes operating at a financial loss, might not be a priority for profit‐generating intervention. The null results for the number of procedures, length of stay, and SMM provide additional evidence for this argument.

At the state level, Medicaid agencies can address the decreased market share issues by increasing Medicaid payments to hospitals, particularly for obstetric services. By adjusting reimbursement rates, states can incentivize hospitals, especially those acquired by PE, to at least maintain services for Medicaid enrollees, particularly in states with low Medicaid‐to‐commercial payment ratios.

At the federal level, the CMS could incorporate PE ownership status into hospital reporting systems and add Medicaid patient volume as a metric in addition to other quality measures. The Federal Trade Commission (FTC) and the Department of Justice (DOJ) could also consider the potential effects of PE hospital acquisitions on Medicaid service provision when evaluating transactions in addition to any anti‐trust concerns.

### 4.1 Limitations

This study has several limitations. First, this study was subject to certain limitations stemming from the data itself. CMS required all states to submit data through the T‐MSIS in 2015 to replace the MAX, but cutover dates vary by state. During this period, some hospitals had reporting issues such as higher‐than‐normal missing diagnosis codes and/or procedure codes. We excluded 4 PE hospitals that were acquired in 2014 or 2015 due to this reporting issue. Data elements availability also changed from MAX to TAF. For example, inpatient claims in MAX have up to 10 diagnosis codes for each record with claims (claims from some states only have 2 in 2011 and 2012), while TAF inpatient claims have up to 13. This results in uneven documentation for many comorbidities and complications that would be worthwhile investigating for patient selection and upcoding. Second, due to the time frame of the study, it did not include the leveraged buyout of HCA by Bain/KKR, which accounts for more than half of all hospitals acquired by PE in history. However, a previous study has examined the hospital behavior changes associated with that PE acquisition extensively [28]. Third, because of the study period and data limitations, our analysis includes only 66 PE‐acquired hospitals, which are fewer than 20% of the 386 PE‐owned hospitals identified as of November 2023 [10]. This limited coverage should be considered when interpreting our findings. Finally, outcomes of the study have been limited to measures from Medicaid claims. Other consequences of PE acquisitions, including payer‐mix change, maternal mortality, waiting time, staffing level, and patient experience, remain unstudied for future research.

## 5 Conclusions

As PE increasingly influences health care delivery in the US, this study is the first to utilize national Medicaid claims data in analyzing its impact on maternal health for Medicaid enrollees. PE acquisition was associated with a 12% decrease in Medicaid obstetric service market share; the decrease is even higher (16%) in states with low Medicaid‐to‐commercial relative prices. There is no evidence of significant changes in C‐section deliveries, patient mix, treatment intensity, and SMM. These findings suggest that state Medicaid agencies have the potential to address these issues by increasing Medicaid payments. This study contributes to the broader PE and health policy literature by drawing attention to the Medicaid population. Our analysis also underscores the importance of monitoring service volumes and availability, especially for the Medicaid population, in addition to any anti‐trust concerns triggered by PE acquisitions.

## Conflicts of Interest

The author declares no conflicts of interest.

## Supporting information


**Appendix S1:** Supporting Information.

## Data Availability

Research data are not shared.
