# Supplementary material for: The Impact of Private Equity Hospital Acquisitions on Maternal Health for Medicaid Patients
Source: Health Serv Res. 2025 Oct 4;61(1):e70048. doi: 10.1111/1475-6773.70048 (PMC12857500; doi:10.1111/1475-6773.70048)

**Appendix**

Table of Contents

# eMethod 1. Exclusion Criteria

eMethod 2. Two-way-fixed-effect Models

## eTable 1. Codes to Identify L&D Claims and Cesarean Deliveries

## eTable 2. Exclusion Codes for Abortion or Born outside of Hospitals

## eTable 3. Codes to Identify High-risk Pregnancy

## eTable 4. Codes to Identify SMM

eTable 5. Changes in Access Associated with PE Acquisition - Subgroup Analysis

eTable 6. Changes in Patient Characteristics Associated with PE Acquisition

eTable 7. Changes in Outcomes with PE acquisition - Subgroup Analyses

## eTable 8. Results from Two-Way-Fixed-Effects Models

## eTable 9. Effect of PE Acquisitions on the Number of Medicaid L&D Hospitalizations

eTable 10. Logit Model Results for 0/1 Outcomes and Patient Selection

## eFigure 1. Flow Diagram for Inclusion and Exclusion

## eFigure 2. Unadjusted Trends for Numbers of L&D Hospitalizations and Market Shares

## **eMethod 1. Exclusion Criteria**

# *Exclusion Criteria for Access Outcome at Hospital-Year Level*

I define hospitals with established obstetric (OB) services as those with an average of 20 or more Medicaid-covered childbirth claims during the pre-acquisition period (2011-2013). Both PE-acquired and control hospitals are excluded if they did not have an established OB service. A reporting issue is identified as if, in a given year, the number of childbirth claims fell below 50% of the average from the year preceding and the year following that year. If a PE-acquired hospital experienced a reporting issue during the acquisition year or the year before, or if that state-year was deemed unusable or of high concern, the PE hospital is excluded from the analysis. Hospitals located in Hospital Referral Regions (HRRs) with only one hospital at any point are also excluded. Hospital-years with reporting issues or in state-years labeled as unusable or of high concern are further excluded from the analysis. Hospital-years with zero Medicaid L&D hospitalizations had the HRR share equal to zero and were included in the analyses.

## *Exclusion Criteria for Hospitalization Level Outcomes*

Hospital-level analyses exclusively incorporate hospitalizations from the treated and matched hospitals. Additionally, patients who are dual-eligible or older than 64 at the time of hospitalization are excluded from the analyses. In the Medicaid fee-for-service (FFS) analysis, claims with zero Medicaid payments are also excluded.

## *State-Year Excluded Due to TAF Data Quality*

| 2014 | DC, KS, NH, RI |
| --- | --- |
| 2015 | DC, KS, MA, OK, PR, VA |
| 2016 | CT, GA, MA, MS, NH, OK, PR, VA |
| 2017 | CT, GA, IA, MA, NH, NY, OK, PR, RI |
| 2018 | CT, GA, MA, NH, NY, OK, PR, RI, VI |
| 2019 | CT, MA, NH, NY, OK, PR, RI |
| 2020 | CT, MA, OK, PR, RI |

##

# **eMethod 2.** **Two-way-fixed-effect Models**

For sensitivity analyses, I conducted OLS two-way fixed effects DiD models following equation (1).

$Y_{it}={\alpha+\beta}_{1}*{PostPE}_{it}+\mu_{i}+\delta_{t}+\varepsilon_{it}$ (1)

where

- $Y_{it}$ is the outcome variable for hospital $i$ at time $t$,
- ${PostPE}_{it}$ is a dummy variable that equals 1 if unit $i$ is in the post-acquisition period at time $t$, and 0 otherwise,
- $\mu_{i}$are unit fixed effects that control for time-invariant differences across units,
- $\delta_{t}$ are time fixed effects at the yearly level that control for shocks common to all units at a given time period,
- $\varepsilon_{it}$is the error term, which is clustered at the hospital level.

In this model, $\beta_{1}$ measures the average treatment effect of PE acquisition on the outcome of interest.

The event study specification is modeled in equation (2).

$Y_{it}={\alpha+\sum_{k,k\neq-1} \beta_{k}EventTime}_{k,it}+\mu_{i}+\delta_{t}+\varepsilon_{it}$ (2)

where ${EventTime}_{k,it}$ is a set of dummy variables for each time period relative to the PE acquisition event, with $k$ representing the number of periods before or after the acquisition for hospital $i$ at time $t$. The other terms are as defined in equation (1). $\beta_{k}$ measures the treatment effect at each relative time period $k$.

Similarly, for outcomes at the hospitalization level including process and quality of care outcomes and payment outcomes, the DiD and event study specifications are modeled in equations (3) and (4), respectively.

$Y_{ijt}={\alpha+\beta}_{1}*{PostPE}_{it}+X_{j}\lambda+\mu_{i}+\delta_{t}+\varepsilon_{ijt}$ (3)

$Y_{ijt}={\alpha+\sum_{k,k\neq-1} \beta_{k}EventTime}_{k,it}+X_{j}\lambda+\mu_{i}+\delta_{t}+\varepsilon_{ijt}$ (4)

where

- $Y_{ijt}$ is the outcome variable for hospitalization $j$at hospital $i$ at time $t$
- $X_{j}$ is a vector of control variables for hospitalization $j$, such as the patient’s age and race ethnicity,
- $\varepsilon_{ijt}$is the error term, which is clustered at the hospital level.

$\mu_{i}$ and $\delta_{t}$ are as defined in equation (1).

## **eTable 1. Codes to Identify L&D Claims and Cesarean Deliveries**

|  | Cesarean | Vaginal | Unknown |
| --- | --- | --- | --- |
| MS-DRG v.33 | 765, 766 | 767, 768, 774, 775 |  |
| MS-DRG v.36 | 783, 784, 785, 786, 787, 788 | 796, 797, 798, 805, 806, 807 |  |
| APR-DRG | 540, 5401, 5402, 5403, 5404 | 541,542,560,5411, 5412, 5413, 5414, 5421, 5422, 5423, 5424, 5601, 5602, 5603, 5604 |  |
| CPT | 59514, 59620 | 59409, 59410, 59612 |  |
| ICD-9-CM PX | 740, 741, 742, 744, 7499 |  | 720, 721, 724, 726, 728, 729, 7221, 7229, 7231, 7239, 7251, 7252, 7253, 7254, 7271, 7279, 7322, 7359, 736 |
| ICD-10-PCS | 10D00Z0, 10D00Z1, 10D00Z2 | 10D07Z3, 10D07Z4, 10D07Z5, 10D07Z6, 10D07Z7, 10D07Z8, 10E0XZZ | 10S07ZZ, 0W8NXZZ |
| ICD-9-CM |  |  | V272, V273, V275, V276, V279, 64420, 64421, 650, V270, V271, V274, V277, Z382 |
| ICD-10-CM | O82, O7582 | O80 | O601, O6010, O6010X0, O6010X1, O6010X2, O6010X3, O6010X4, O6010X5, O6010X9, O6012, O6012X0, O6012X1, O6012X2, O6012X3, O6012X4, O6012X5, O6012X9, O6013, O6013X0, O6013X1, O6013X2, O6013X3, O6013X4, O6013X5, O6013X9, O6014, O6014X0, O6014X1, O6014X2, O6014X3, O6014X4, O6014X5, O6014X9, Z370, Z371, Z372, Z373, Z374, Z3750, Z3751, Z3752, Z3753, Z3754, Z3759, Z3760, Z3761, Z3762, Z3763, Z3764, Z3769, Z377, Z379 |

Notes: Enrollees must be at least 9.

## **eTable 2. Exclusion Codes for Abortion or Born outside of Hospitals**

|  | Codes |
| --- | --- |
| CPT | 59120, 59121, 59130, 59135, 59136, 59140, 59150, 59151, 59840, 59841, 59850, 59851, 59852, 59855, 59856, 59857, 59812, 59820, 59821, 59830 |
| ICD-9-CM PX | 743, 6901, 6951, 7491, 750 |
| ICD-10-PCS | 10A00ZZ, 10A03ZZ, 10A04ZZ, 10A07ZX, 10A07ZZ, 10A07ZZ, 10A08ZZ, 10A08ZZ |
| ICD-9-CM | 63300, 63301, 63310, 63311, 63320, 63321, 63380, 63381, 63390, 63391, 630, 6310, 6318, 632 |
| ICD-10-CM | O00101, O00102, O00109, O00111, O00112, O00119, O00201, O00202, O00209, O00211, O00212, O00219, O0080, O0081, O0090, O0091, O019, O021, O0289, A34, Z371 |

## **eTable3. Codes to Identify High-risk Pregnancy**

|  | Codes |
| --- | --- |
| ICD-9-CM | 042, V08, 642.6, 642.61, 642.62, 642.63, 648.51, 648.52, 648.53, 648.54, 648.6, 648.61, 648.62, 648.63, 648.64, 644.2, 644.21, 646, 646.01, 646.03, 656.4, 656.41, 656.43, V27.1, V27.3, V27.4, V27.7, 652.21, 669.6, 669.61, 652.31, 652.41, 652.7, 652.71, 660.3, 660.31, O64.0XX0, 653.6, 653.61, 653.63, 653.71, 655.01, 678.1, 678.11, 678.13, 641.01, 641.11, 654.2, 654.21, 654.23, O34.211, O34.212, O34.219, 654.3, 654.31, 654.32, 654.33, 654.34, 663, 663.01, 663.03, 663.5, 663.51, 663.53, 665, 665.01, 665.03, 665.1, 665.11, 665.12, 665.14, 660.7, 660.71, 660.73, 651, 651.01, 651.03, V91.00, V91.01, V91.02, V91.03, V91.09, 651.1, 651.11, 651.13, V91.10, V91.11, V91.12, V91.19, 651.2, 651.21, 651.23, V91.20, V91.22, V91.29, V91.90, 651.3, 651.31, 651.33, 651.4, 651.41, 651.43, 651.5, 651.51, 651.53, 651.6, 651.61, 651.63, 651.7, 651.71, 651.73, 651.8, 651.81, 651.83, 651.9, 651.91, 651.93, 652.6, 652.61, 652.63, V27.2, V27.5, V27.6, V91.21, V91.91, V91.92, V91.99, 660.5, 660.51, 660.53, 761.5, 662.3, 662.31, 662.33 |
| ICD-10-CM | B20, O98.711, O98.712, O98.713, O98.719, O98.72, O98.73, Z21, O15.00, O15.9, O15.02, O15.03, O15.1, O15.2, O14.20, O14.22, O14.23, O14.24, O99.411, O99.412, O99.413, O99.42, Z87.74, O99.43, O99.419, O10.22, O10.32, O22.50, O22.51, O22.52, O22.53, O60.10X0, O60.12X0, O60.13X0, O60.14X0, Z3A.08, Z3A.09, Z3A.10, Z3A.11, Z3A.12, Z3A.13, Z3A.14, Z3A.15, Z3A.16, Z3A.17, Z3A.18, Z3A.19, Z3A.20, Z3A.21, Z3A.22, Z3A.23, Z3A.24, Z3A.25, Z3A.26, Z3A.27, Z3A.28, Z3A.29, Z3A.30, Z3A.31, Z3A.32, Z3A.33, Z3A.34, Z3A.35, Z3A.36, O31.00X0, O31.01X0, O31.02X0, O31.03X0, O36.4XX0, Z37.1, Z37.3, Z37.4, Z37.7, O32.1XX0, O64.1XX0, O32.2XX0, O64.4XX0, O64.5XX0, O32.3XX0, O64.2XX0, O64.3XX0, O33.6XX0, O33.7, O35.0XX0, O30.029, O30.021, O30.022, O30.023, O43.212, O43.213, O43.219, O43.222, O43.223, O43.229, O43.232, O43.233, O43.239, O44.00, O44.02, O44.03, O44.10, O44.12, O44.13, O44.20, O44.22, O44.23, O44.30, O44.32, O44.33, O44.40, O44.42, O44.43, O44.50, O44.52, O44.53, O45.002, O45.003, O45.009, O45.012, O45.013, O45.019, O45.022, O45.023, O45.029, O45.092, O45.093, O45.099, O34.21, O34.513, O69.0XX0, O69.4XX0, O71.00, O71.02, O71.03, O71.1, O66.41, O66.5, O30.009, O30.019, O30.039, O30.049, O30.099, O30.001, O30.002, O30.003, O30.011, O30.012, O30.013, O30.031, O30.032, O30.033, O30.041, O30.042, O30.043, O30.091, O30.092, O30.093, O30.109, O30.119, O30.129, O30.199, O30.101, O30.102, O30.103, O30.111, O30.112, O30.113, O30.121, O30.122, O30.123, O30.191, O30.192, O30.193, O30.209, O30.229, O30.299, O30.201, O30.202, O30.203, O30.211, O30.212, O30.213, O30.221, O30.222, O30.223, O30.291, O30.292, O30.293, O31.10X0, O31.10X1, O31.10 |

## **eTable 4. Codes to Identify SMM**

|  | Codes |
| --- | --- |
| ICD-10 DX | I21.xx, I22.x, I71.xx, I79.0, N17.x, O90.4, J80, J95.1, J95.2, J95.3, J95.82x, J96.0x, J96.2x, J96.9x, R06.03, R09.2, O88.112, O88.113, O88.119, O88.12, O88.13, I46.x, I49.0x, D65, D68.8, D68.9, O45.002, O45.003, O45.009, O45.012, O45.013, O45.019, O45.022, O45.023, O45.029, O45.092, O45.093, O45.099, O46.002, O46.003, O46.009, O46.012, O46.013, O46.019, O46.022, O46.023, O46.029, O46.092, O46.093, O46.099, O67.0, O72.3, O15.x, I97.120, I97.121, I97.130, I97.131, I97.710, I97.711, A81.2, G45.x, G46.x, G93.49, H34.0x, I60.xx, I61.xx, I62.xx, I63.00, I63.01x, I63.1xx, I63.2xx, I63.3xx, I63.4xx, I63.5xx, I63.6, I63.8x, I63.9, I65.xx, I66.xx, I67.xx, I68.xx, O22.50, O22.52, O22.53, I97.810, I97.811, I97.820, I97.821, O87.3, I50.1, I50.20, I50.21, I50.23, I50.30, I50.31, I50.33, I50.40, I50.41, I50.43, I50.810, I50.811, I50.813, I50.814, I50.82, I50.83, I50.84, I50.89, I50.9, J81.0, O29.112, O29.113, O29.114, O29.115, O29.116, O29.117, O29.118, O29.119, O29.122, O29.123, O29.124, O29.125, O29.126, O29.127, O29.128, O29.129, O29.192, O29.193, O29.194, O29.195, O29.196, O29.197, O29.198, O29.199, O29.212, O29.213, O29.214, O29.215, O29.216, O29.217, O29.218, O29.219, O29.292, O29.293, O29.294, O29.295, O29.296, O29.297, O29.298, O29.299, O74.0, O74.1, O74.2, O74.3, O89.0x, O89.1, O89.2, T88.2XXA, T88.3XXA, A32.7, A40.x, A41.x, I76, O85, O86.04, R65.20, R65.21, T81.12XA, T81.44XA, O75.1, R57.x, T78.2XXA, T81.10XA, T81.11XA, T81.19XA, T88.6XXA, D57.00, D57.01, D57.02, D57.211, D57.212, D57.219, D57.411, D57.412, D57.419, D57.811, D57.812, D57.819, I26.x, O88.012, O88.013, O88.019, O88.02, O88.03, O88.212, O88.213, O88.219, O88.22, O88.23, O88.312, O88.313, O88.319, O88.32, O88.33, O88.812, O88.813, O88.819, O88.82, O88.83, T80.0XXA |
| ICD-10 PX | 5A12012, 5A2204Z, 30230K0, 30230L0, 30230M0, 30230N0, 30230P0, 30230R0, 30230T0, 30230H1, 30230K1, 30230L1, 30230M1, 30230N1, 30230P1, 30230R1, 30230T1, 30233H0, 30233K0, 30233L0, 30233M0, 30233N0, 30233P0, 30233R0, 30233T0, 30233H1, 30233K1, 30233L1, 30233M1, 30233N1, 30233P1, 30233R1, 30233T1, 30240H0, 30240K0, 30240L0, 30240M0, 30240N0, 30240P0, 30240R0, 30240T0, 30240H1, 30240K1, 30240L1, 30240M1, 30240N1, 30240P1, 30240R1, 30240T1, 30243H0, 30243K0, 30243L0, 30243M0, 30243N0, 30243P0, 30243R0, 30243T0, 30243H1, 30243K1, 30243L1, 30243M1, 30243N1, 30243P1, 30243R1, 30243T1, 0UT90ZL, 0UT90ZZ, 0UT97ZL, 0UT97ZZ, 0B110F4, 0B113F4, 0B114F4, 5A1935Z, 5A1945Z, 5A1955Z |
| ICD-9 DX | 410.xx, 441.xx, 584.5, 584.6, 584.7, 584.8, 584.9, 669.3x, 518.5x, 518.81, 518.82, 518.84, 799.1, 673.1x, 427.41, 427.42, 427.5, 286.6, 286.9, 641.3x, 666.3x, 642.6x, 997.1, 046.3, 348.39, 362.34, 430.xx, 431.xx, 432.xx, 433.xx, 434.xx, 435.xx, 436.xx, 437.xx, 671.5x, 674.0x, 997.02, 428.0, 428.1, 428.20, 428.21, 428.23, 428.30, 428.31, 428.33, 428.40, 428.41, 428.43, 428.9, 518.4, 668.0x, 668.1x, 668.2x, 995.4, 995.86, 038.xx, 449, 785.52, 995.91, 995.92, 998.02, 670.2x, 669.1x, 785.50, 785.51, 785.59, 995.0, 998.0, 998.00, 998.01, 998.09, 282.42, 282.62, 282.64, 282.69, 289.52, 415.0, 415.1x, 673.0x, 673.2x, 673.3x, 673.8x |
| ICD-9 PX | 68.39, 68.49, 68.59, 68.69, 68.79, 68.9, 68.4, 68.5, 68.6, 68.7, 68.3, 31.1, 99.6x, 96.70, 96.71, 96.72 |

**eTable 5. Changes in access associated with PE acquisition - subgroup analysis**

|  | Ownership | | Hospital size | |
| --- | --- | --- | --- | --- |
|  | For-profit | Non-profit | Small | Large |
| PE acquisition effect | -0.007 | -0.021^*^ | -0.011^*^ | -0.017 |
|  | [ -0.018, 0.003] | [-0.042, -0.000] | [ -0.022,-0.000] | [-0.044, 0.001] |
| Pre-acquisition outcome, mean (SD) | 0.09 (0.13) | 0.17 (0.22) | 0.11 (0.16) | 0.14 (0.17) |
| N | 2,020 | 788 | 1,814 | 994 |

*Notes*: Small hospitals are defined as average bed size of less than 100. For-profit and non-profit status are defined using pre-acquisition data. Government-run hospitals are grouped with non-profit hospitals. All non-profit hospitals were converted to for-profit status after acquisition. Coefficients are estimated using the Callaway & Sant’Anna method described in Method. Standard errors are clustered at the hospital level. Brackets contain 95% confidence intervals. The average of pre-acquisition market share for PE hospitals is included to aid in the interpretation of the magnitude. ^*^ indicates p<0.05. SD=Standard Deviation.

# **eTable 6. Changes in Patient Characteristics Associated with PE Acquisition**

|  | Advanced age | Non-white |
| --- | --- | --- |
| PE baseline outcome, mean (SD) | 0.17 (0.30) | 0.61 (0.49) |
| Coefficient | 0.003 | -0.013 |
|  | [-0.004, 0.009] | [-0.043, 0.018] |
| N | 1,106,414 | 1,106,414 |

*Notes:* Advanced age is defined as greater than 35 at admission.

Coefficients are estimated using the Callaway & Sant’Anna method described

in section 3. Standard errors are clustered at the hospital level. Brackets contain 95% confidence intervals. ^*^ indicates p<0.05. SD=Standard Deviation.

# **eTable 7. Changes in Outcomes with PE Acquisition - Subgroup Analysis**

|  | Low-risk  C-section | No. Procedures | Length of Stay | SMM per 1000* |
| --- | --- | --- | --- | --- |
| *Panel A: By Age Group* | | | | |
| Advanced Age |  |  |  |  |
| PE baseline outcome,  mean (SD) | 0.369 (0.48) | 2.87 (1.09) | 2.32 (1.00) | 2.02 (44.9) |
| Coefficient | -0.033 | -0.02 | -0.05 | 1.20 |
|  | [-0.073, 0.007] | [-0.148, 0.117] | [-0.168, 0.075] | [-11.22, 13.61] |
| N | 88,328 | 88,551 | 88,551 | 40,751 |
| Non-Advanced Age |  |  |  |  |
| PE baseline outcome,  mean (SD) | 0.256 (0.48) | 3.00 (1.19) | 2.48 (1.12) | 1.59 (39.8) |
| Coefficient | -0.01 | -0.02 | -0.05 | -0.3 |
|  | [-0.035,0.022] | [-0.13,0.085] | [-0.129, 0.027] | [-4.85, 4.28] |
| N | 1,016,200 | 1,017,165 | 1,017,165 | 412,819 |
| *Panel B: By Racial Group* | | | | |
| Non-White |  |  |  |  |
| PE baseline outcome,  mean (SD) | 0.266 (0.44) | 2.87 (1.11) | 2.39 (1.02) | 1.76 (41.9) |
| Coefficient | -0.03 | -0.03 | -0.05 | -1.5 |
|  | [-0.057,0.004] | [-0.143,0.085] | [-0.173,0.062] | [-5.94, 2.93] |
| N | 674,349 | 675,349 | 675,349 | 283,930 |
| White |  |  |  |  |
| PE baseline outcome,  mean (SD) | 0.261 (0.44) | 3.13 (1.25) | 2.25 (1.00) | 1.49 (38.7) |
| Coefficient | 0.02 | 0.02 | -0.01 | 0.3 |
|  | [-0.005,0.043] | [-0.12,0.156] | [-0.090, 0.078] | [-6.63, 7.27] |
| N | 430,183 | 430,367 | 430,367 | 169,640 |

## **eTable 8. Result from Two-Way-Fixed-Effects Models**

| Outcomes | Coefficient | Confidence Interval | N |
| --- | --- | --- | --- |
| Access |  |  |  |
| HRR share: Overall | -0.017* | [-0.027, -0.008] | 2,808 |
| HRR share: High Relative Price | -0.008 | [-0.021, 0.005] | 476 |
| HRR share: Low Relative Price | -0.018* | [-0.032, -0.003] | 1,258 |
| HRR share: Small Hospitals | -0.014* | [ -0.026, -0.003] | 2,020 |
| HRR share: Large Hospitals | -0.022* | [-0.040, -0.005] | 788 |
| HRR share: For-profit Hospitals | -0.013* | [-0.026, -0.003] | 1,814 |
| HRR share: Non-profit Hospitals | -0.023* | [-0.037, -0.010] | 994 |
| Patient Selectin: Advanced Age | -0.026 | [-0.008, 0.002] | 1,106,414 |
| Patient Selectin: Non-white | -0.027 | [-0.082, 0.028] | 1,106,414 |
| Process and Quality of Care |  |  |  |
| Low-risk C-section | -0.02 | [-0.075, 0.027] | 1,035,295 |
| No. Procedures | -0.14 | [-0.292, 0.015] | 1,106,414 |
| Length of Stay | -0.08 | [-0.19, 0.02] | 1,106,414 |
| SMM per 1000^+^ | 0.88 | [-5.03, 3.26] | 426,912 |

*Notes:*^+^ Due to differences in data structure between MAX and TAF inpatient claims, it's important to note that MAX claims only contain ten diagnosis codes (some states in earlier years only have two), whereas TAF claims could include up to thirteen. As a result, the SMM rate is not directly comparable between earlier years in MAX and years in TAF. Therefore, the SMM analysis only includes hospitals that were acquired in 2018 using data from 2016 to 2020. ^*^ indicates p<0.05

## **eTable 9. Effect of PE Acquisitions on the Number of Medicaid L&D Hospitalizations**

|  | Poisson Model | OLS |
| --- | --- | --- |
| Mean (SD) | 454 (309.95) | |
| Coefficients | -0.05 | -41.8 |
| CI | [-0.06, -0.04] | [-77.65, -5.95] |

*Notes:* The number of Medicaid L&D hospitalizations was right winsorized at 99%.

## **eTable 10. Logit Model Results for 0/1 Outcomes and Patient Selection**

|  | Low-risk  C-section | SMM | Advanced Age | Non-white |
| --- | --- | --- | --- | --- |
| Marginal Effect | -0.009 | 0.001 | 0 .006 | 0.044 |
| CI | [-0.012, -0.006] | [-0.001,0.003] | [0.003,0.009] | [0.039,0.050] |

*Notes:* Logit models cannot converge with hospital-level fixed effects, thus only contain year fixed effects and other covariates.

## **eFigure 1. Flow Diagram for Inclusion and Exclusion**


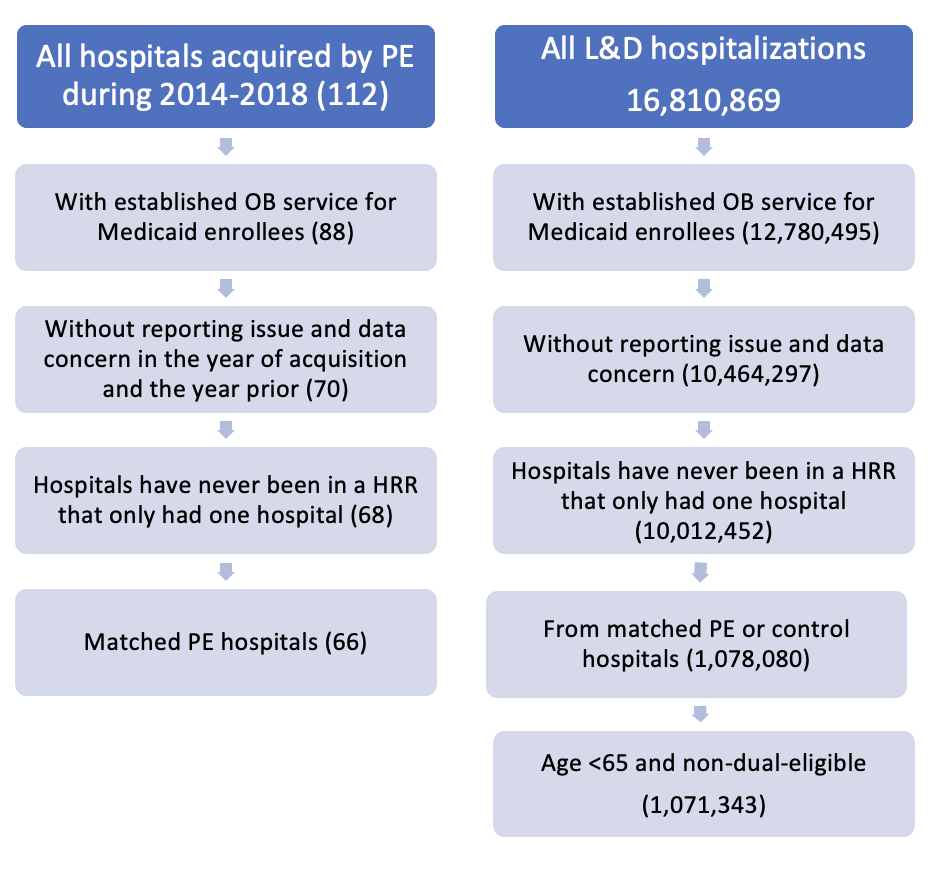


## **eFigure 2. Unadjusted Trends for Numbers of L&D Hospitalizations and Market Shares**


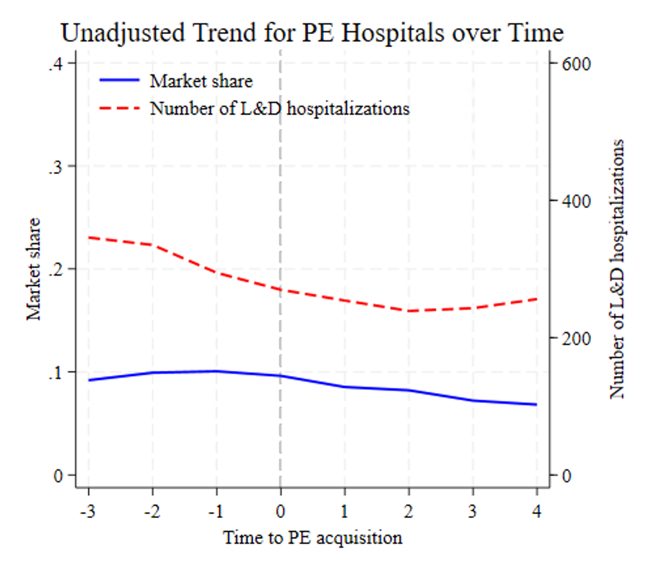

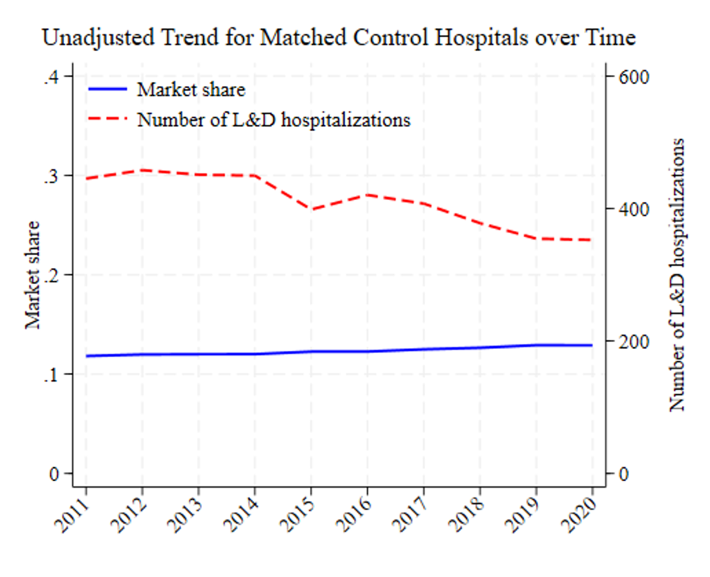

Supplement: Supplementary file 1 — Appendix S1: Supporting Information. [file HESR-61-0-s001.docx]
